# Relationship between Maternal and First Year of Life Dispensations of Antibiotics and Antiasthmatics

**DOI:** 10.3390/antibiotics7030084

**Published:** 2018-09-17

**Authors:** Guro Haugen Fossum, Morten Lindbæk, Svein Gjelstad, Kari J. Kværner

**Affiliations:** 1 Antibiotic Center for Primary Care, Department of General Practice, Institute of Health and Society, University of Oslo, P.O. Box 1130 Blindern, N-0318 Oslo, Norway; morten.lindbak@medisin.uio.no (M.L.); svein.gjelstad@medisin.uio.no (S.G.); 2 C3-Centre for Connected Care, Oslo University Hospital, N-0424 Oslo, Norway; uxkakv@ous-hf.no; 3 BI Norwegian Business School, 0484 Oslo, Norway

**Keywords:** antibacterial agents, pregnancy, children, asthma medication, pharmacoepidemiology

## Abstract

Antibiotics are the most frequent prescription drugs used by pregnant women. Our objective was to investigate if the dispensation of antibiotics and antiasthmatics in children less than 1 year of age is associated with prenatal antibiotic exposure. A secondary aim was to explore the incidence of dispensed antibiotics in pregnancy and dispensed antibiotics and antiasthmatics in children. We conducted an observational study using the Peer Academic Detailing study database to select patients eligible for match in the Medical Birth Registry of Norway, a total of 7747 mother-and-child pairs. Details on antibiotic and antiasthmatic pharmacy dispensations were obtained from the Norwegian Prescription Database. One quarter (1948 of 7747) of the mothers in the study had been dispensed antibiotics during pregnancy. In their first year of life, 17% (1289) of the children had had an antibiotic dispensation, 23% (1747) an antiasthmatic dispensation, and 8% (619) of the children had had both. We found a significant association between dispensed antibiotics in pregnancy and dispensed antibiotics to the child during their first year of life; OR = 1.16 (95% CI: 1.002–1.351). The association was stronger when the mothers were dispensed antibiotics at all, independent of the pregnancy period; OR = 1.60 (95% CI: 1.32–1.94). We conclude that the probability for dispensation of antibiotics was increased in children when mothers were dispensed antibiotics, independent of pregnancy. Diagnostic challenges in the very young and parental doctor-seeking behavior may, at least in part, contribute to the association between dispensations in mothers and children below the age of one year.

## 1. Introduction

Antibiotics are the most frequent prescription drugs used by pregnant women [1]. A Dutch study showed that 20.8% of women were prescribed antibiotics during their pregnancy [2]. Similar numbers are found in a study from Canada [3]. A Norwegian study from 2004–2006 found as many as 43% of first-time pregnant women received at least one antibiotic prescription in the time interval three months prior to until three months after pregnancy [1]. Different types of penicillin represent more than 70% of these antibiotics dispensed [1].

Possible long-term effects of prenatal antibiotic exposure are of interest. The outcome of atopic diseases, including atopic dermatitis and asthma, are well-studied. A Danish study published in 2013 found an increased risk of asthma exacerbation in a prospective study of 411 infants where the mothers were exposed to antibiotics in the third trimester [4]. The risk increase was confirmed in a national birth cohort, where asthma hospitalization and inhaled corticosteroids were used as outcomes [4]. Other studies also found antibiotic use in the third trimester of pregnancy to be associated with a small increase in the risk of asthma in preschool children, after adjusting for confounding variables [5,6]. A study from Italy in 2016, with wheezing as the outcome measure, found an association with third-trimester antibiotic exposure [7].

Respiratory tract infections, RTIs, are the main cause for antibiotic prescriptions in children [8]. The general practitioner (GP) faces a dilemma when evaluating small children presenting with acute respiratory symptoms. He or she relies on a range of factors when assessing children with respiratory symptoms, including the parents’ assessment of the condition [9]. A British study showed that most children receiving antibiotics for respiratory symptoms in general practice actually have a viral infection, and that RTIs were caused by viral pathogens in 77% of cases considered more serious than a common cold [10]. The former and current Norwegian guidelines for antibiotic prescriptions in general practice address the challenges in diagnostics and precise recommendations on when to prescribe to children with lower RTIs [11,12]. Deciding when wheezing as part of an RTI is a clinical sign of childhood asthma is difficult, particularly in children aged 0–2 years [13]. The European Respiratory Society Task Force 2008 report states that most cases of wheezing in preschool children are episodic and triggered by viral infections, and suggests not to use the term asthma to describe wheezing illness in preschool children [14]. Few studies address the youngest children and most rely on diagnoses given by doctors and not the total use of medication, including the combination of antibiotics and antiasthmatics.

The aim of this study was to investigate the association between prenatal exposure to antibiotics and receiving an antibiotic or antiasthmatic prescription among children aged younger than one year. A secondary aim was to explore the incidence of dispensed antibiotics in pregnancy and dispensed antibiotics and antiasthmatics in children.

## 2. Results

### 2.1. Dispensations

Of the 7747 mothers in the database, 1948 (25%) had an antibiotic prescription dispensed during pregnancy. During their first two years of life, 3755 (49%) and 3668 (47%) of the children had a prescription for antibiotics (J01) or antiasthmatics (R03) dispensed, respectively. Further characteristics of the cohort are shown in Table 1.

**Figure 1:**
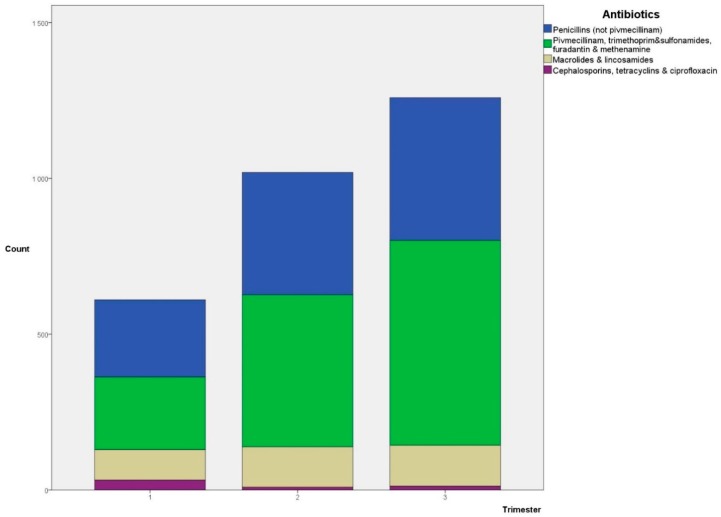
Chart showing the distribution of different antibiotics dispensed to pregnant women according to trimesters.

During pregnancy, 2888 antibiotic prescriptions were dispensed (*n* = 1948). The distribution of different types of antibiotics according to pregnancy trimesters is shown in Figure 1. Penicillins and antibiotics typically used for urinary tract infections (UTIs) are the largest two groups in all three trimesters (penicillins: 41%, 39%, and 36%; and UTI antibiotics: 38%, 48%, and 52%, for trimesters 1, 2, and 3, respectively). 

A total of 1937 antibiotic (J01) and 3230 antiasthmatic (R03) prescriptions were dispensed to children during their first year of life (*n* = 2417). The vast majority of the antibiotics were penicillin V, 62%, and 25% were macrolides. Of the antiasthmatics prescribed, 32% were short-term beta-2 antagonists, 20% were inhalation steroids or combination drugs, and 48% were ephedrine. Dispensations of multiple prescriptions of antiasthmatics during the first year were registered in 649 children (8% of the total 7747, 37% of children with at least one antiasthmatic prescription during the first year).

### 2.2. Regression Analysis

**Figure 2:**
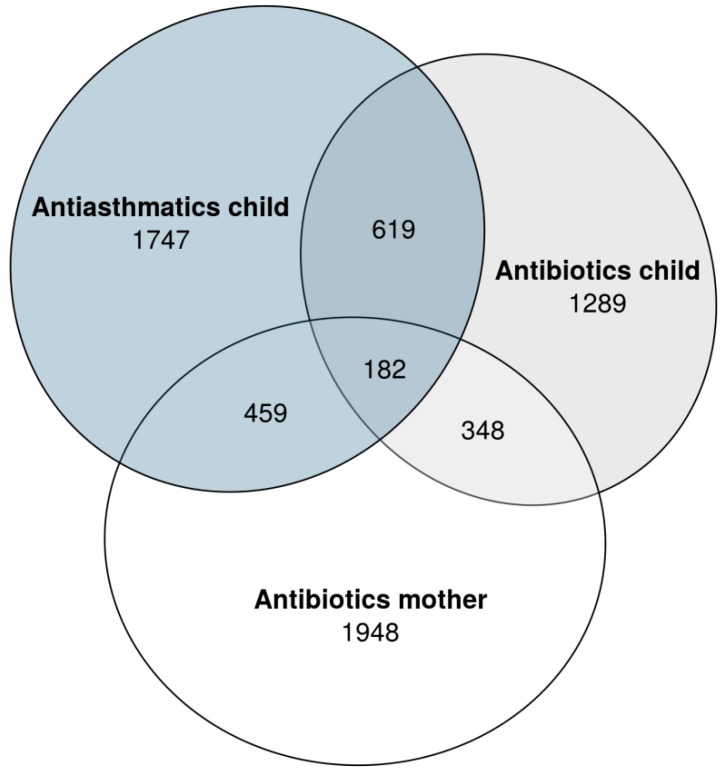
The number of women prescribed antibiotics in pregnancy and the number of children prescribed antibiotics/antiasthmatics during the first year of life [15].

During the first year of life, 1289 (17%) of the children had an antibiotic prescription dispensed and 1747 (23%) had a prescription for antiasthmatics dispensed. These numbers in relation to the number of mothers with antibiotics prescribed during pregnancy is shown in Figure 2. The association between antibiotics dispensed and antiasthmatics dispensed in the first year was high; OR = 4.37 (95% CI: 3.85–4.96).

Using a logistic regression model, we found a significant association between dispensed antibiotics in pregnancy and dispensed antibiotics for the child aged younger than 1 year; OR = 1.16 (95% CI: 1.002–1.351). The association was more clear when the antibiotic was dispensed during the third trimester; OR = 1.27 (95% CI: 1.06–1.53). A similar association was found, although not significant, when looking at dispensed antiasthmatics for children aged younger than 1 year (Table 2). The association was stronger in the group of children having both medications dispensed (*n* = 619), OR = 1.43 (95% CI: 1.12–1.82) (Table 2).

When the association between maternal antibiotic dispensation, independent of pregnancy period, and dispensations in children was assessed, a significant relationship was found both for antibiotics and antiasthmatics: OR = 1.60 (95% CI: 1.32–1.94) for children having antibiotics dispensed, OR = 1.32 (95% CI: 1.12–1.55) for children having antiasthmatics dispensed, and OR = 1.64 (95% CI: 1.25–2.17) for children having both medications dispensed, respectively. Correspondingly, for maternal dispensation of antibiotics outside the pregnancy period (4394 mothers), the association only remained significant for children having antibiotics dispensed; OR = 1.15 (95% CI: 1.002–1.311).

In the children born in 2005, 4533 in total, with a follow-up time of more than two years, dispensations after 2 years of age were assessed. In total, 685 of 1279 (54%) of the children with antiasthmatics dispensed during their first year also had dispensations after age 2 years. Logistic regression showed an OR of 2.59 (95% CI: 2.27–2.96), adjusted for gender and prematurity. Of 917 (20% of 2005-born children) children who had antibiotics dispensed before 1 year of age, 416 (45%) had antiasthmatics dispensed when older than 2 years; OR = 1.55 (95% CI: 1.34–1.79), also adjusted for gender and prematurity.

## 3. Discussion

Our main findings were that one in four women had an antibiotic dispensed during pregnancy and that dispensation of antibiotics and antiasthmatics for children during their first year of life was common: 17% (1289) and 23% (1747), respectively. There was an increased probability for both antibiotic and antiasthmatic dispensations during the first year of life related to maternal antibiotic dispensations, independent of the pregnancy period.

Our database recruitment is based on patient appointments or contacts with their GP; thus, a selection bias of doctor-seeking patients cannot be excluded. Our study population found less children than mothers with a match in the Medical Birth Registry. Accordingly, we suspect some children may not have been appointed with their GP prior to the age of 2 years, while others only have had out-of-hours service contacts. If these children differ from our study cohort, it may have influenced the generalizability of our findings. The Peer Academic Detailing, Rx-PAD, study has been previously shown to be representative of Norwegian general practice, additionally we have important information such as the mother’s smoking status, gestational week of birth, and the child’s gender [16]. However, possible confounders such as socioeconomic status, breastfeeding, siblings, and birth weight cannot be completely ruled out.

Using patients from the Rx-PAD database only allowed for dispensations registered in the time 2004–2007. More recent data would be preferable; however, a Norwegian study from 2018 on prescription drugs in pregnancy from 2005–2015 reports an increase in use of all medication from 57% in 2005/6 to 62% in 2014/15, due to other medications than antibiotics [17].

Data from the Norwegian Prescription Database, NorPD, does not include diagnostic information, and we are therefore not able to discuss the indication for the antibiotic and antiasthmatic treatments prescribed. We know that most antibiotics and antiasthmatics are prescribed by GPs [18,19], but prescription habits may change over time and are influenced by several factors unaccounted for in this study. The dispensation of antibiotics in pregnancy shown in Table 1, with 7%, 11%, and 13% in the respective trimesters, corresponds well with a previous study from Norway in the same time period using NorPD, showing 9.5%, 12%, and 12.6% of women receiving dispensed antibiotics in the three respective trimesters of pregnancy [1]. We found that 23% of children had a prescription of antibiotics dispensed during their first year of life, and as many as 48.5% when aged 0–2 years. A recent publication from Norway showed a lower number in 2016, where 38.9% aged 0–2 years had at least one antibiotic prescription dispensed, but numbers for this age group from 2004 to 2009 exceeds 50%, corresponding with our findings [8].

There are several studies addressing the prescription of antiasthmatics to children, but few single out the youngest, aged younger than 1 year. A GP database study by Zuidgeest et al. published in 2008 reported that 7.5% children below 18 years were prescribed antiasthmatics, with 11.7% in the age group 0–2 years [20]. Schokker et al. found an 8% prescription rate of antiasthmatics in primary care for children aged 0–9 years [21]. Our findings show that 23% of children had antiasthmatic medication dispensed (R03) during the first year of life. However, these numbers include dispensations of ephedrine. Looking at R03 dispensations without ephedrine, the number of children is 714 (9.2%), which is more aligned with the two Dutch studies. Data from Norway which includes children aged 0–4 between 2004 and 2015 show a median of 10.4% for R03 dispensations excluding ephedrine [19].

We found that more than half the children with antiasthmatics dispensed in the first year also had antiasthmatics dispensed after age 2 years. A Dutch study including 165 children with antiasthmatics dispensed in the first year of life found that 58.8% keep using such medications after the first prescription, but that only 10.3% continue for the next three years [22]. Our study also showed a positive association between antibiotic dispensation prior to age 1 year and antiasthmatics after age 2 years. In a case control study by Metsälä et al., an association of antibiotic use by children under 1 year of age and later development of asthma was found, with the odds ratio increasing with number of antibiotic purchases [23]. The use of antiasthmatic prescription data as a proxy for asthma in children was studied by Furu et al. in 2007, concluding that it gives a valid prevalence estimate of ongoing asthma [24]. The variance in antiasthmatic prescriptions by GPs is greatest in the youngest children [25], although variation in GPs’ antibiotic prescription patterns for RTIs in preschool children was also found [26]. Furthermore, the etiology of lower RTIs are mostly viral, accounting for as much as 63% of the diagnosed pneumonias [27].

Our study shows an increased probability of dispensation of antibiotics alone or in combination with antiasthmatics to children when exposed to antibiotics during pregnancy, and the association was stronger when the mothers were dispensed antibiotics at all, independent of the pregnancy period. Whether this is related to doctor-seeking behavior or increased illness cannot be distinguished based on these data. The association between antibiotic exposure in pregnancy and asthma in preschool children has been shown in several studies [4,5,6,7]. Our findings are aligned with the latter, although our estimates were not significant when looking at dispensed antiasthmatics alone, only in combination with antibiotics. A U.S. study from 2011 reported an association between antibiotic exposure before 6 months of age and asthma and allergy at 6 years of age [28]. One of the suggested explanations was the difficulty in distinguishing symptoms of asthma and respiratory tract infections in young children. Other studies link antibiotic exposure with childhood infections, such as otitis media [29]. The association between maternal antibiotic dispensation and dispensation to the child in the first year of life, independent of the pregnancy period, is shown in two large Scandinavian studies by Örtqvist et al. and by Stokholm et al. in 2014, and our results are aligned with their findings [30,31].

Research indicate that parents’ experience, confidence, and efficacy influence the likelihood of consulting for RTIs [32]. Consequently, another possible explanation for the association of dispensed antibiotics to mothers and subsequently to the child may be the continuation of the same doctor-seeking behavior when the woman becomes a parent. A study from Hong Kong found that parents’ health-seeking behavior on behalf of their children is identical to their own preferences, although to a lesser extent regarding antibiotics [33]. A Norwegian study found that inhabitants’ consultations rates have as much impact on the number of RTI antibiotics prescribed as do the GPs’ prescription rates [34]. Studies on parental concerns and expectations show that symptomatic relief is a major concern, but parents do not necessarily expect an antibiotic and would often prefer an alternative nonantibiotic approach [35].

## 4. Materials and Methods

**Figure 3:**
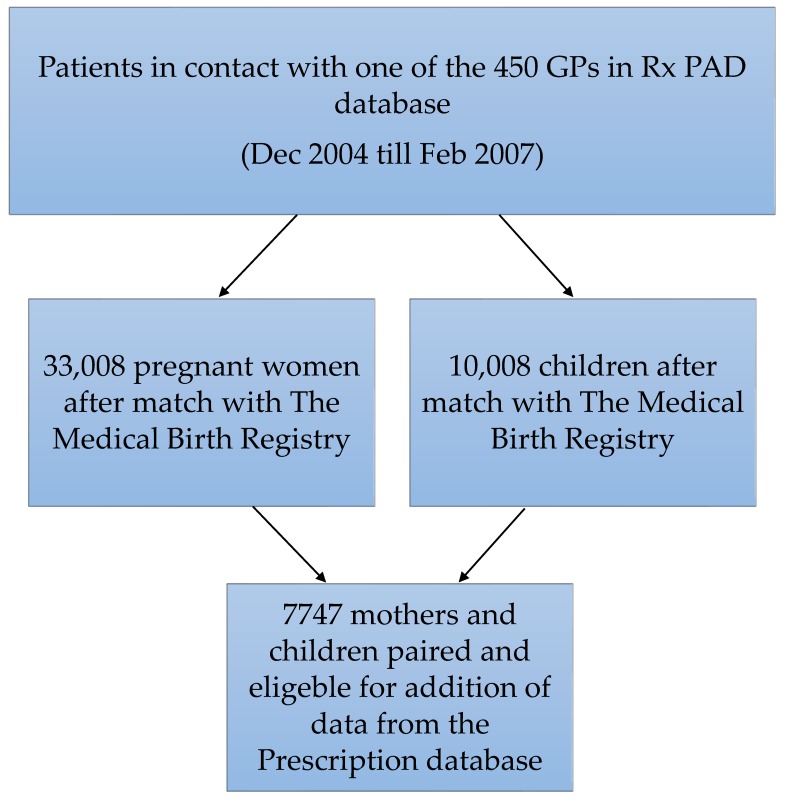
Selection of patients. (GP = general practitioner). Trimesters are calculated by length of pregnancy according to Norwegian standards published previously [1,40]. Preterm birth is defined as birth before 37 gestational weeks, according to WHO and Norwegian standards. Mother’s smoking habits are based on data from the Medical Birth Registry of Norway, as are gender of the child and mother’s age. Antibiotics are defined as all ATC code J01 prescriptions. Antiasthmatics are defined as all ATC code R03 prescriptions.

This cohort consists of matching mothers and children, first selected by contact with their GP between December 2004 until February 2007. The contact ensured inclusion in our Rx-PAD database consisting of more than 450 GPs and their patients [36]. The inclusion in the Rx-PAD database ended in 2007 and has provided data in order to study several aspects of antibiotic prescribing in general practice [16,26,37,38,39]. Women with a match in the Medical Birth Registry of Norway and a matching child were included in the final cohort. The initial match before pairing the mothers and their child gave about 33,000 pregnant women and 10,000 children. Further pairing gave us a total of 7747 mother/child pairs. The large number of unpaired mothers and children can probably be explained by one party visiting another doctor than their regular GP, by only seeing a doctor out-of-hours, or by not having had any contact with their GP at all during the study period. The selection of patients can be seen in Figure 3.

Prescription data was added from the Norwegian Prescription database. The prescriptions are categorized according to the Anatomical Therapeutic Chemical Classification System (ATC). Data on dispensed medication to both mothers and children include all antibiotics (ATC code J01) and respiratory medication (ATC code R03).

Outcome measures are based on registered antiasthmatics (R03) or antibiotics (J01) dispensed at pharmacy within the first year of life. Antibiotic exposure during pregnancy is used overall and divided into trimesters. Odds ratios are used to explore the associations between antibiotic exposure in pregnancy and antibiotics and/or antiasthmatics dispensed to the child. The level of significance was set at *p* < 0.05. Factors corrected for are: gender, mothers’ age, mothers’ smoking, and preterm birth. In the logistic regression model, we did not include antiasthmatics when antibiotics was the outcome and vice versa. This was due to the suspected clinical overlap between the two treatments in children with respiratory problems.

## 5. Conclusions

We found an increased probability of having medication for respiratory problems dispensed to children less than 1 year of age when their mothers have been dispensed antibiotics. This probability was independent of the time of prescription and may not be directly related to pregnancy, and thus external factors which may contribute to this association would be of interest to explore. Consideration of diagnostic challenges, specifically the differentiation between infectious and obstructive symptoms in the very young patients, and doctor-seeking behavior by parents on behalf of their children, are of particular interest to consider.

## Figures and Tables

**Table 1:** Background information about the cohort of mothers and children.

Mothers and Children: *n* = 7747	
Mothers’ mean age (*n* = 7746) in years (SD)	30.48 ± 4.9
Mothers’ median age in years (range)	31 (15–46)
Antibiotic prescription in pregnancy (*n* = 7747) n (%)	1948 (25.1%)
First trimester *n* (%)	511 (6.6%)
Second trimester *n* (%)	822 (10.6%)
Third trimester *n* (%)	1014 (13.1%)
Mothers’ asthma *n* (%)	295 (3.8%)
Smoking in pregnancy (*n* = 6310) *n* (%)	953 (15.1%)
Length of pregnancy (mean days ± SD)	277 ± 13
Preterm birth *n* (%)	535 (6.9%)
Female-gender child (*n* = 7737) *n* (%)	3668 (47.4%)
Antibiotic prescription for child	-
First year of life *n* (%)	1289 (16.6%)
First two years of life *n* (%)	3755 (48.5%)
Antiasthmatic prescription for child	-
First year of life *n* (%)	1747 (22.6%)
First two years of life *n* (%)	3668 (47.3%)

**Table 2:** Estimated crude odds ratios (cOR) and adjusted odds ratios (aOR) with 95% confidence intervals between antibiotic exposure in pregnancy and dispensed antibiotics and/or dispensed antiasthmatics during the first year of life. (Statistically significant ORs in bold, *p* < 0.05).

Time of Dispension	Antibiotics in First Year (*n* = 1289)		Antiasthmatics in First Year (*n* = 1747)		Antibiotics and Antiasthmatics in First Year (*n* = 619)	
	cOR	Aor *	cOR	aOR *	cOR	aOR *
Antibiotics in Pregnancy	1.12 (0.98–1.29)	**1.16 (1.00–1.35) ^1^**	1.08 (0.96–1.22)	1.06 (0.92–1.21)	**1.27 (1.06–1.52)**	**1.30 (1.06–1.58)**
Trimester 1 Trimester 2 Trimester 3	0.85 (0.66–1.10) 1.05 (0.87–1.27) **1.27 (1.08–1.51)**	0.93 (0.71–1.22) 1.16 (0.94–1.43) **1.27 (1.06–1.53)**	**0.72 (0.57–0.91)** 1.10 (0.93–1.31) **1.19 (1.02–1.38)**	**0.73 (0.56–0.94)** 1.06 (0.88–1.28) 1.18 (0.99–1.40)	0.81 (0.57–1.16) 1.24 (0.96–1.59) **1.45 (1.17–1.81)**	0.84 (0.57–1.25) **1.37 (1.05–1.79)** **1.43 (1.12–1.82)**

* Adjusted for gender, prematurity, mother’s smoking, and mother’s age. ^1^ The OR is marked significant as the full CI: 1.002–1.351, *p* = 0.047.
